# Draft Genome Sequence of the Antimony-Oxidizing *Pseudomonas* sp. Strain SbOxS1, Isolated from Stibnite Mine Tailing Soil

**DOI:** 10.1128/MRA.01218-20

**Published:** 2020-12-03

**Authors:** Natsuko Hamamura, Nobuyoshi Nakajima, Shigeki Yamamura

**Affiliations:** a Department of Biology, Faculty of Science, Kyushu University, Fukuoka, Japan; b Center for Environmental Biology and Ecosystem Studies, National Institute for Environmental Studies, Tsukuba, Ibaraki, Japan; c Center for Regional Environmental Research, National Institute for Environmental Studies, Tsukuba, Ibaraki, Japan; University of Southern California

## Abstract

*Pseudomonas* sp. strain SbOxS1 was isolated from stibnite mine tailing soil for its ability to oxidize antimonite. We present a draft genome sequence of strain SbOxS1, which contains 6,484 predicted protein-coding sequences. This genome information extends our understanding of the physiological versatility of antimony-transforming microorganisms.

## ANNOUNCEMENT

Antimony (Sb) is a naturally occurring toxic metalloid. Although the concentrations of Sb in soils are generally low, elevated levels of Sb have been released via mining and other anthropogenic activities (1). Despite its toxicity, microorganisms have developed mechanisms to tolerate and catalyze redox transformations of Sb (2–5). *Pseudomonas* sp. strain SbOxS1, previously designated strain S1, was isolated from stibnite mine tailing soil for its ability to oxidize antimonite (6).

Strain SbOxS1 was cultured from a mine tailing soil collected in Ehime, Japan (33°53′N, 133°12′E), using the extinction dilution technique (6) and grown in minimal Xm medium (7) with 0.002% (wt/vol) yeast extract and 100 μM Sb(III) (as potassium antimonyl tartrate). DNA was extracted using a MoBio PowerSoil DNA isolation kit (Qiagen), following the manufacturer’s protocol. A paired-end library (with an average insert size of 350 bp) was prepared by using a NEBNext Ultra DNA library prep kit (New England BioLabs), and genome sequencing was performed on the HiSeq X sequencing platform (Illumina, CA, USA) at the National Institute for Environmental Studies. Overall, 7,814,926 raw paired-end reads were generated (2 × 150 bp), and low-quality sequences (Q ≤ 13) were removed using the Trim_Reads tool implemented in CLC Genomics Workbench v20.0.2 (GW; Qiagen). Sequences were *de novo* assembled in slow mode in GW with default parameters except for the minimum contig length (500 bp) and word size (30). The resulting 99 contigs had an *N*_50_ value of 316,253 bp and a maximum contig length of 742,657 bp. The draft genome sequence of strain SbOxS1 was 7,358,650 bp long with 184.0× genome coverage and a G+C content of 60.1%. Annotation was conducted using the NCBI Prokaryotic Genome Annotation Pipeline (8) and Prokka v1.12 via KBase (9) with default parameters, resulting in 6,484 predicted protein-coding sequences, 63 tRNAs, and 6 complete rRNAs (1 copy each of 16S and 23S and 4 copies of 5S). BLASTn analysis of the 16S rRNA gene showed that this strain is closely related to other *Pseudomonas* strains (sequence identity of >99%), such as Pseudomonas jessenii strain CIP 105274 (GenBank accession number NR_024918.1) and Pseudomonas vancouverensis strain DhA-51 (NR_041953.1).

The functional annotation of the coding DNA sequences (CDSs) indicated the presence of two *ars* operons associated with As and Sb resistance and the arsenite oxidase gene *aioAB*, although this strain was unable to oxidize arsenite under the conditions previously examined (6). Additionally, the draft genome sequences contained functional genes encoding nitrate reductase and enzymes associated with the aerobic degradation of aromatic hydrocarbons such as phenol hydroxylase, benzoate 1,2-dioxygase, and catechol 1,2-dioxyganse. The draft genome sequence of *Pseudomonas* sp. strain SbOxS1 provides valuable information regarding the physiological versatility of antimony-metabolizing microorganisms.

### Data availability.

This draft genome sequence was deposited in GenBank under accession number JAAVXC000000000, BioProject accession number PRJNA622630, BioSample accession number SAMN14523789, and SRA accession number SRR11467866.
